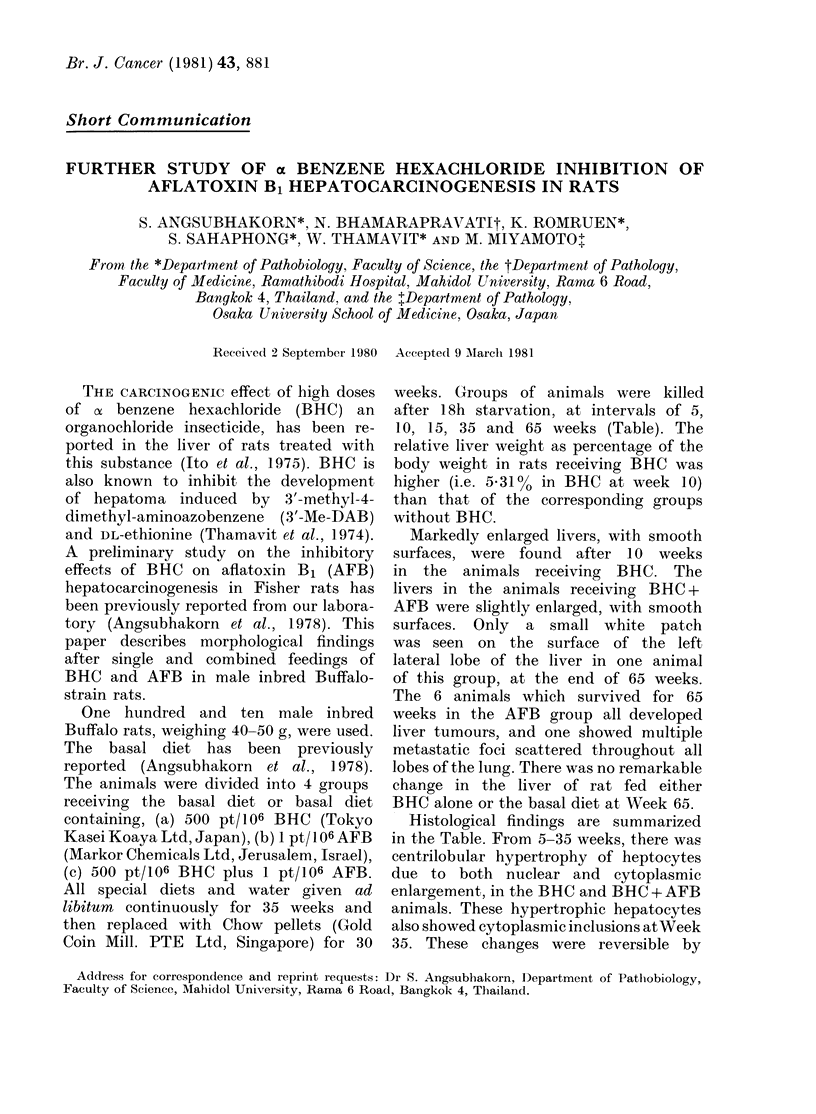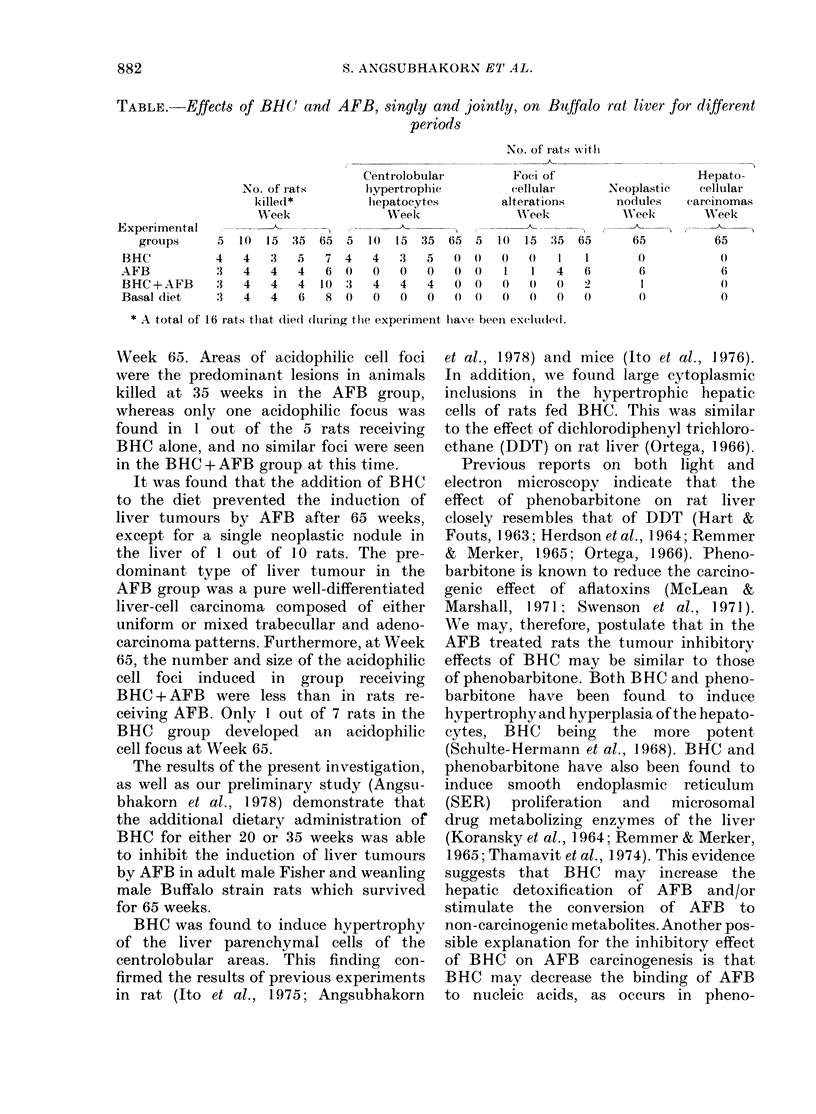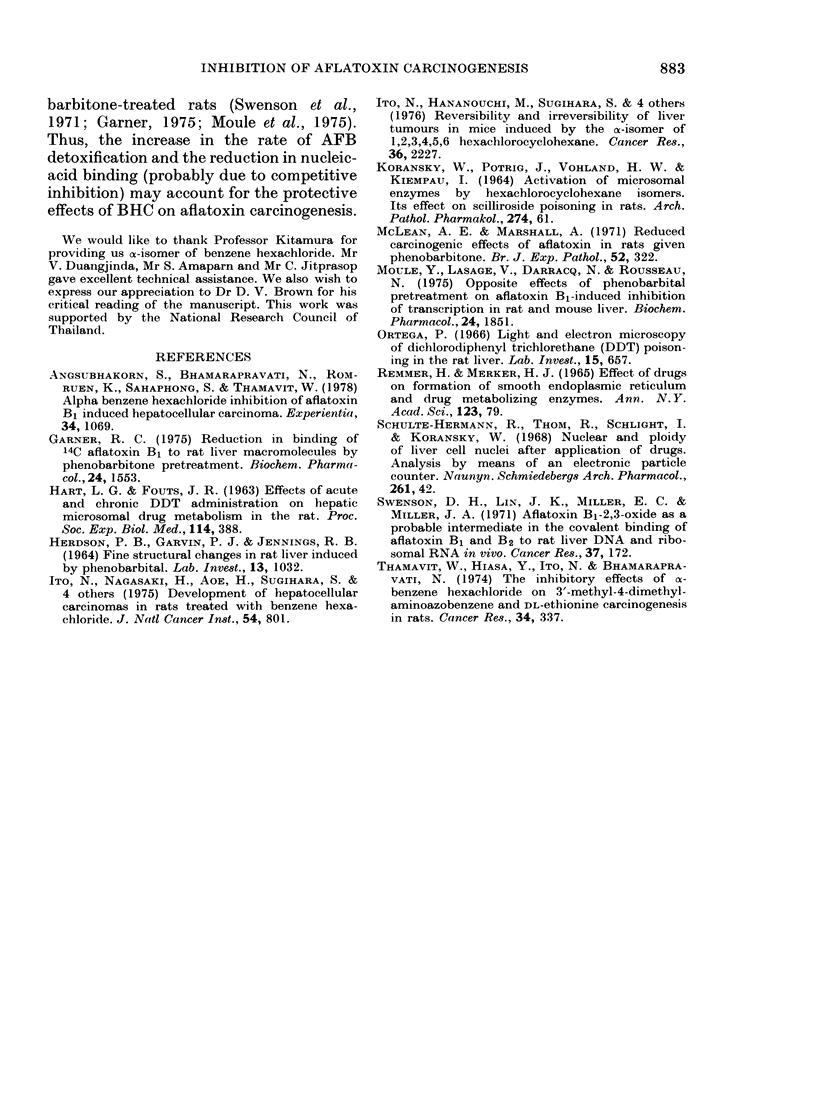# Further study of alpha benzene hexachloride inhibition of aflatoxin B1 hepatocarcinogenesis in rats.

**DOI:** 10.1038/bjc.1981.129

**Published:** 1981-06

**Authors:** S. Angsubhakorn, N. Bhamarapravati, K. Romruen, S. Sahaphong, W. Thamavit, M. Miyamoto


*Br. J. Cancer* (1981) **43**, 881

***Short Communication***

### FURTHER STUDY OF α BENZENE HEXACHLORIDE INHIBITION OF AFLATOXIN $B_1$ HEPATOCARCINOGENESIS IN RATS

S. ANGSUBHAKORN*, N. BHAMARAPRAVATI†, K. ROMRUEN*, S. SAHAPHONG*, W. THAMAVIT* AND M. MIYAMOTO‡

*From the *Department of Pathobiology, Faculty of Science, the †Department of Pathology, Faculty of Medicine, Ramathibodi Hospital, Mahidol University, Rama 6 Road, Bangkok 4, Thailand, and the ‡Department of Pathology, Osaka University School of Medicine, Osaka, Japan*

Received 2 September 1980 Accepted 9 March 1981

THE CARCINOGENIC effect of high doses of α benzene hexachloride (BHC) an organochloride insecticide, has been reported in the liver of rats treated with this substance (Ito *et al.*, 1975). BHC is also known to inhibit the development of hepatoma induced by 3′-methyl-4-dimethyl-aminoazobenzene (3′-Me-DAB) and DL-ethionine (Thamavit *et al.*, 1974). A preliminary study on the inhibitory effects of BHC on aflatoxin $B_1$ (AFB) hepatocarcinogenesis in Fisher rats has been previously reported from our laboratory (Angsubhakorn *et al.*, 1978). This paper describes morphological findings after single and combined feedings of BHC and AFB in male inbred Buffalo-strain rats.

One hundred and ten male inbred Buffalo rats, weighing 40–50 g, were used. The basal diet has been previously reported (Angsubhakorn *et al.*, 1978). The animals were divided into 4 groups receiving the basal diet or basal diet containing, (a) 500 pt/$10^6$ BHC (Tokyo Kasei Koaya Ltd, Japan), (b) 1 pt/$10^6$ AFB (Markor Chemicals Ltd, Jerusalem, Israel), (c) 500 pt/$10^6$ BHC plus 1 pt/$10^6$ AFB. All special diets and water given *ad libitum* continuously for 35 weeks and then replaced with Chow pellets (Gold Coin Mill. PTE Ltd, Singapore) for 30 weeks. Groups of animals were killed after 18h starvation, at intervals of 5, 10, 15, 35 and 65 weeks (Table). The relative liver weight as percentage of the body weight in rats receiving BHC was higher (i.e. 5·31% in BHC at week 10) than that of the corresponding groups without BHC.

Markedly enlarged livers, with smooth surfaces, were found after 10 weeks in the animals receiving BHC. The livers in the animals receiving BHC+AFB were slightly enlarged, with smooth surfaces. Only a small white patch was seen on the surface of the left lateral lobe of the liver in one animal of this group, at the end of 65 weeks. The 6 animals which survived for 65 weeks in the AFB group all developed liver tumours, and one showed multiple metastatic foci scattered throughout all lobes of the lung. There was no remarkable change in the liver of rat fed either BHC alone or the basal diet at Week 65.

Histological findings are summarized in the Table. From 5–35 weeks, there was centrilobular hypertrophy of heptocytes due to both nuclear and cytoplasmic enlargement, in the BHC and BHC+AFB animals. These hypertrophic hepatocytes also showed cytoplasmic inclusions at Week 35. These changes were reversible by

Address for correspondence and reprint requests: Dr S. Angsubhakorn, Department of Pathobiology, Faculty of Science, Mahidol University, Rama 6 Road, Bangkok 4, Thailand.

TABLE.—*Effects of BHC and AFB, singly and jointly, on Buffalo rat liver for different periods*

| | No. of rats with | | | | | | | | | | | | | | | | | |
|---|---|---|---|---|---|---|---|---|---|---|---|---|---|---|---|---|---|---|
| | No. of rats killed* Week | | | | | Centrolobular hypertrophic hepatocytes Week | | | | | Foci of cellular alterations Week | | | | | Neoplastic nodules Week | Hepato-cellular carcinomas Week |
| Experimental groups | 5 | 10 | 15 | 35 | 65 | 5 | 10 | 15 | 35 | 65 | 5 | 10 | 15 | 35 | 65 | 65 | 65 |
| BHC | 4 | 4 | 3 | 5 | 7 | 4 | 4 | 3 | 5 | 0 | 0 | 0 | 0 | 1 | 1 | 0 | 0 |
| AFB | 3 | 4 | 4 | 4 | 6 | 0 | 0 | 0 | 0 | 0 | 0 | 1 | 1 | 4 | 6 | 6 | 6 |
| BHC+AFB | 3 | 4 | 4 | 4 | 10 | 3 | 4 | 4 | 4 | 0 | 0 | 0 | 0 | 0 | 2 | 1 | 0 |
| Basal diet | 3 | 4 | 4 | 6 | 8 | 0 | 0 | 0 | 0 | 0 | 0 | 0 | 0 | 0 | 0 | 0 | 0 |

* A total of 16 rats that died during the experiment have been excluded.

Week 65. Areas of acidophilic cell foci were the predominant lesions in animals killed at 35 weeks in the AFB group, whereas only one acidophilic focus was found in 1 out of the 5 rats receiving BHC alone, and no similar foci were seen in the BHC+AFB group at this time.

It was found that the addition of BHC to the diet prevented the induction of liver tumours by AFB after 65 weeks, except for a single neoplastic nodule in the liver of 1 out of 10 rats. The predominant type of liver tumour in the AFB group was a pure well-differentiated liver-cell carcinoma composed of either uniform or mixed trabecullar and adenocarcinoma patterns. Furthermore, at Week 65, the number and size of the acidophilic cell foci induced in group receiving BHC+AFB were less than in rats receiving AFB. Only 1 out of 7 rats in the BHC group developed an acidophilic cell focus at Week 65.

The results of the present investigation, as well as our preliminary study (Angsubhakorn *et al.*, 1978) demonstrate that the additional dietary administration of BHC for either 20 or 35 weeks was able to inhibit the induction of liver tumours by AFB in adult male Fisher and weanling male Buffalo strain rats which survived for 65 weeks.

BHC was found to induce hypertrophy of the liver parenchymal cells of the centrolobular areas. This finding confirmed the results of previous experiments in rat (Ito *et al.*, 1975; Angsubhakorn *et al.*, 1978) and mice (Ito *et al.*, 1976). In addition, we found large cytoplasmic inclusions in the hypertrophic hepatic cells of rats fed BHC. This was similar to the effect of dichlorodiphenyl trichloroethane (DDT) on rat liver (Ortega, 1966).

Previous reports on both light and electron microscopy indicate that the effect of phenobarbitone on rat liver closely resembles that of DDT (Hart & Fouts, 1963; Herdson *et al.*, 1964; Remmer & Merker, 1965; Ortega, 1966). Phenobarbitone is known to reduce the carcinogenic effect of aflatoxins (McLean & Marshall, 1971; Swenson *et al.*, 1971). We may, therefore, postulate that in the AFB treated rats the tumour inhibitory effects of BHC may be similar to those of phenobarbitone. Both BHC and phenobarbitone have been found to induce hypertrophy and hyperplasia of the hepatocytes, BHC being the more potent (Schulte-Hermann *et al.*, 1968). BHC and phenobarbitone have also been found to induce smooth endoplasmic reticulum (SER) proliferation and microsomal drug metabolizing enzymes of the liver (Koransky *et al.*, 1964; Remmer & Merker, 1965; Thamavit *et al.*, 1974). This evidence suggests that BHC may increase the hepatic detoxification of AFB and/or stimulate the conversion of AFB to non-carcinogenic metabolites. Another possible explanation for the inhibitory effect of BHC on AFB carcinogenesis is that BHC may decrease the binding of AFB to nucleic acids, as occurs in phenobarbitone-treated rats (Swenson *et al.*, 1971; Garner, 1975; Moule *et al.*, 1975). Thus, the increase in the rate of AFB detoxification and the reduction in nucleic-acid binding (probably due to competitive inhibition) may account for the protective effects of BHC on aflatoxin carcinogenesis.

We would like to thank Professor Kitamura for providing us α-isomer of benzene hexachloride. Mr V. Duangjinda, Mr S. Amaparn and Mr C. Jitprasop gave excellent technical assistance. We also wish to express our appreciation to Dr D. V. Brown for his critical reading of the manuscript. This work was supported by the National Research Council of Thailand.

### REFERENCES

ANGSUBHAKORN, S., BHAMARAPRAVATI, N., ROMRUEN, K., SAHAPHONG, S. & THAMAVIT, W. (1978) Alpha benzene hexachloride inhibition of aflatoxin $B_1$ induced hepatocellular carcinoma. *Experientia*, **34**, 1069.

GARNER, R. C. (1975) Reduction in binding of $^{14}C$ aflatoxin $B_1$ to rat liver macromolecules by phenobarbitone pretreatment. *Biochem. Pharmacol.*, **24**, 1553.

HART, L. G. & FOUTS, J. R. (1963) Effects of acute and chronic DDT administration on hepatic microsomal drug metabolism in the rat. *Proc. Soc. Exp. Biol. Med.*, **114**, 388.

HERDSON, P. B., GARVIN, P. J. & JENNINGS, R. B. (1964) Fine structural changes in rat liver induced by phenobarbital. *Lab. Invest.*, **13**, 1032.

ITO, N., NAGASAKI, H., AOE, H., SUGIHARA, S. & 4 others (1975) Development of hepatocellular carcinomas in rats treated with benzene hexachloride. *J. Natl Cancer Inst.*, **54**, 801.

ITO, N., HANANOUCHI, M., SUGIHARA, S. & 4 others (1976) Reversibility and irreversibility of liver tumours in mice induced by the α-isomer of 1,2,3,4,5,6 hexachlorocyclohexane. *Cancer Res.*, **36**, 2227.

KORANSKY, W., POTRIG, J., VOHLAND, H. W. & KIEMPAU, I. (1964) Activation of microsomal enzymes by hexachlorocyclohexane isomers. Its effect on scilliroside poisoning in rats. *Arch. Pathol. Pharmakol.*, **274**, 61.

MCLEAN, A. E. & MARSHALL, A. (1971) Reduced carcinogenic effects of aflatoxin in rats given phenobarbitone. *Br. J. Exp. Pathol.*, **52**, 322.

MOULE, Y., LASAGE, V., DARRACQ, N. & ROUSSEAU, N. (1975) Opposite effects of phenobarbital pretreatment on aflatoxin $B_1$-induced inhibition of transcription in rat and mouse liver. *Biochem. Pharmacol.*, **24**, 1851.

ORTEGA, P. (1966) Light and electron microscopy of dichlorodiphenyl trichlorethane (DDT) poisoning in the rat liver. *Lab. Invest.*, **15**, 657.

REMMER, H. & MERKER, H. J. (1965) Effect of drugs on formation of smooth endoplasmic reticulum and drug metabolizing enzymes. *Ann. N.Y. Acad. Sci.*, **123**, 79.

SCHULTE-HERMANN, R., THOM, R., SCHLIGHT, I. & KORANSKY, W. (1968) Nuclear and ploidy of liver cell nuclei after application of drugs. Analysis by means of an electronic particle counter. *Naunyn. Schmiedebergs Arch. Pharmacol.*, **261**, 42.

SWENSON, D. H., LIN, J. K., MILLER, E. C. & MILLER, J. A. (1971) Aflatoxin $B_1$-2,3-oxide as a probable intermediate in the covalent binding of aflatoxin $B_1$ and $B_2$ to rat liver DNA and ribosomal RNA *in vivo*. *Cancer Res.*, **37**, 172.

THAMAVIT, W., HIASA, Y., ITO, N. & BHAMARAPRAVATI, N. (1974) The inhibitory effects of α-benzene hexachloride on 3′-methyl-4-dimethylaminoazobenzene and DL-ethionine carcinogenesis in rats. *Cancer Res.*, **34**, 337.